# Caffeine Consumption Prevents Diabetes-Induced Memory Impairment and Synaptotoxicity in the Hippocampus of NONcZNO10/LTJ Mice

**DOI:** 10.1371/journal.pone.0021899

**Published:** 2012-04-13

**Authors:** João M. N. Duarte, Paula M. Agostinho, Rui A. Carvalho, Rodrigo A. Cunha

**Affiliations:** 1 Center for Neuroscience and Cell Biology (CNC), University of Coimbra, Coimbra, Portugal; 2 Department of Life Sciences, Faculty of Sciences and Technology, University of Coimbra, Coimbra, Portugal; 3 Faculty of Medicine, University of Coimbra, Coimbra, Portugal; Universidad Federal de Santa Catarina, Brazil

## Abstract

Diabetic conditions are associated with modified brain function, namely with cognitive deficits, through largely undetermined processes. More than understanding the underlying mechanism, it is important to devise novel strategies to alleviate diabetes-induced cognitive deficits. Caffeine (a mixed antagonist of adenosine A_1_ and A_2A_ receptors) emerges as a promising candidate since caffeine consumption reduces the risk of diabetes and effectively prevents memory deficits caused by different noxious stimuli. Thus, we took advantage of a novel animal model of type 2 diabetes to investigate the behavioural, neurochemical and morphological modifications present in the hippocampus and tested if caffeine consumption might prevent these changes. We used a model closely mimicking the human type 2 diabetes condition, NONcNZO10/LtJ mice, which become diabetic at 7–11 months when kept under an 11% fat diet. Caffeine (1 g/l) was applied in the drinking water from 7 months onwards. Diabetic mice displayed a decreased spontaneous alternation in the Y-maze accompanied by a decreased density of nerve terminal markers (synaptophysin, SNAP25), mainly glutamatergic (vesicular glutamate transporters), and increased astrogliosis (GFAP immunoreactivity) compared to their wild type littermates kept under the same diet. Furthermore, diabetic mice displayed up-regulated A_2A_ receptors and down-regulated A_1_ receptors in the hippocampus. Caffeine consumption restored memory performance and abrogated the diabetes-induced loss of nerve terminals and astrogliosis. These results provide the first evidence that type 2 diabetic mice display a loss of nerve terminal markers and astrogliosis, which is associated with memory impairment; furthermore, caffeine consumption prevents synaptic dysfunction and astrogliosis as well as memory impairment in type 2 diabetes.

## Introduction

Reduced peripheral glucose regulation and diabetic conditions affect the central nervous system, contributing to diabetic encephalopathy [1]. In particular, diabetic individuals display an augmented incidence of cognitive problems, which are particularly associated with atrophy of the hippocampal formation, which is involved in learning and memory processing [2], [3]. However, the mechanisms underlying the development of diabetic encephalopathy and associated cognitive impairments remain unknown.

The prevention of neuronal dysfunction and neurodegeneration represents a major goal of medical research, and an emerging candidate to manage diabetes-induced neurodegeneration is caffeine [4]. Caffeine is the most widely consumed psychoactive substance and acts as an antagonist of adenosine A_1_ receptors (A_1_R) and A_2A_R at non-toxic doses [5]. Caffeine consumption alleviates cognitive impairment in both humans and animals [6], [7], namely in Alzheimer’s disease [8]–[10], and affords protection upon CNS injury [11], [12]. Furthermore, several studies indicate that habitual coffee consumption reduces the risk of diabetes [13]. Conversely, we found that streptozotocin-induced diabetes modifies the expression and density of adenosine receptors in the hippocampus [14], as occurs in most noxious brain conditions [11] and caffeine prevents streptozotocin-induced neurotoxicity [15], [16]. Thus, we hypothesize that chronic caffeine treatment could prevent the alterations of neuropathology and function associated with the hippocampus known to occur in type 2 diabetes.

We now took advantage of a new mouse strain modelling type 2 diabetes, NONcNZO10/LtJ mice [17], to test if chronic caffeine consumption prevents the diabetes-induced molecular and morphological alterations in the hippocampus. NONcNZO10/LtJ mice are a recombinant congenic strain generated by combining trait loci from New Zealand Obese (NZO/HlLt) and non-obese non-diabetic (NON/LtJ) mice [18]; NONcNZO10/LtJ mice display maturity onset obesity (after 13 weeks, under higher fat diets) associated with a diabetes phenotype characterised by mild hyperglycemia and insulin resistance in skeletal muscle, liver and heart [17], [18]; the polygenic nature and the relatively mild obesity of this model closely resemble human type 2 diabetes, thus making it an attractive model to investigate modifications of brain morphology and function and to test if caffeine might indeed affect diabetes-associated brain dysfunction. We obtained the first evidence that these diabetic mice display a loss of nerve terminal markers, namely of glutamatergic markers, together with an evident astrogliosis, which are associated with a decreased short-term memory performance. Further re-enforcing this association between synaptotoxicity and astrogliosis with memory impairment, we found that the prolonged consumption of caffeine abrogated the diabetes-induced synaptotoxicity and astrogliosis and preserved memory function.

## Materials and Methods

### Ethics Statement

All animals used in the study were handled according to EU guidelines (86/609/EEC); all mice were deeply anesthetized under halothane atmosphere before sacrifice by decapitation. The experiments were approved by the Center’s Ethics committee (Comissão de Instalação do Biotério FMUC-CNC; permit issued on 7^th^ September 2007).

### Animals and Caffeine Treatment

Male NONcNZO10/LtJ mice and control NON/LtJ mice ( Jackson Laboratory, Bar Harbor, Maine, USA) were maintained under an 11% fat diet from the age of 7 months onwards. Half of the mice from each group were randomly selected for treatment with 1 g/L caffeine (Sigma-Aldrich, Sintra, Portugal) supplied in the drinking water during 20 weeks (from 30 to 50 weeks of age). This dose of caffeine was selected based on its previously reported ability to prevent memory impairment and modifications of hippocampal synaptic proteins in other animal models of brain disorders [15], [19]. It corresponds to a high intake of caffeine, leading to a concentration of caffeine in the plasma and brain parenchyma similar to that expected to occur in humans consuming 8 cups of coffee daily [20]. This high intake of caffeine was chosen also because it is such a high intake, rather than low or moderate intake, of coffee that attenuate the burden of diabetes [13]. Weight and water/caffeine consumption were monitored during the treatment and pre-prandial glycaemia was measured monthly from tail blood [14], [15]. Therefore, 4 groups of mice were used throughout the study: control NON/LtJ mice (10 animals), control NON/LtJ mice treated with caffeine (10 animals), diabetic NONcNZO10/LtJ mice (10 animals), diabetic NONcNZO10/LtJ mice treated with caffeine (9 animals). Mice in the control and diabetic groups were randomly assigned to consume either water or caffeine before beginning of the study. All these animals were used for general characterization (see Table 1) and behavioural assays and, within each group, 4 mice were used for immunohistochemical analysis and 5–6 different mice were used for immunocytochemical and neurochemical analysis.

**Table 1:** Characteristics of the mice involved in the study, during and/or after caffeine treatment.

	Control mice	Diabetic mice	Diabetic mice + Caffeine	Control mice + Caffeine
**Caffeine intake** (mg/day/kg)				
month 1			101±12	91±8
month 2			104±5	92±5
month 3			91±5	87±5
month 4			86±5	85±4
**Serum caffeine** (µM)			50.1±13.8	54.8±10.4
**Body weight** (g)				
before	43.6±1.5	46.1±1.0	47.0±1.4 $	41.4±0.7
month 1	44.9±1.4	50.1±0.9	45.7±1.5	43.7±0.9 #
month 2	48.7±1.4	51.1±1.3	46.8±1.2	46.7±1.9
month 3	50.4±1.7	52.5±1.6	47.9±1.2	46.6±1.6 #
month 4	52.9±2.3	53.5±2.2	47.5±1.2 #	47.7±1.9 #
**Glycaemia** (mg/dL)				
month 1	138.7±3.6	375.0±57.9 ***	249.6±47.4 #	138.4±7.7 ###
month 2	165.6±6.0	396.9±55.2 ***	259.9±41.7 #	181.2±17.8 ###
month 3	153.4±11.9	357.9±58.2 ***	261.4±34.1 #	150.0±7.3 ###
month 4	162.67±23.8	426.1±61.9 ***	282.3±22.9 #. $	139.4±11.2 ###
**Serum insulin** (µg/L)	6.2±1.2	33.4±12.0 *	38.9±7.9 **	13.2±3.9

The study included 10 mice in the control, diabetic and caffeine groups, and 9 diabetic mice treated with caffeine (1 g/L in drinking water), which were 7 months of age at the beginning of the study. Data are mean±SEM. * P<0.05, ** P<0.01, *** P<0.001 compared to control; # P<0.05, ### P<0.001 compared to diabetic; $ P<0.05 compared to caffeine.

### Quantification of Serum Insulin and Caffeine

At the end of the 20-week caffeine treatment, plasma insulin concentration was quantified by enzyme immunoassay [14], [15]. Serum caffeine concentration was evaluated, as previously described [15], by HPLC using a reverse-phase column [LiChroCART 125×4 mm LiChrospher 100 RP-18 (5 µm) cartridge fitted into a ManuCART holder (Merck, Darmstadt, Germany)] and a Gilson system equipped with a UV detector set at 274 nm. The eluent was 40% (v/v) methanol with a flow rate of 0.8 mL/min. Identification and quantification of caffeine was performed by comparison of relative retention time with standards (1–100 µM).

### Behavioural Tasks

Locomotor activity was determined using an open field arena after habituation [10], [15]. Spontaneous alternation was assessed in a Y-maze to access hippocampal-dependent memory, constituting a basic mneumonic task, which does not involve an evident ‘learning’ component and does not isolate memory performance but is a robust predictor of short term memory performance [10], [15], [19]. The total number of entries in the arms of the Y maze also allows accessing locomotor activity and exploratory behaviour. All the mice were run in the Y maze before the initiation of the treatments (high fat or caffeine or respective controls) and then tested again after the 4 month treatment, the day after the open field test. Clearly, the use of a single behavioural 1 day paradigm only allows a rough estimate of short memory performance; however, previous studies from our group using rodent models of Alzheimer’s disease have shown that the Y maze test has sufficient sensitivity to highlight insidious changes of memory performance (see [10], [21]), with the great advantage of being devoid of conditioning or aversive components. Never the less, it should be made clear that this experimental design is only intended to provide evidence for the existence of putative impairments of short term memory performance rather than allowing to define if such deficits also occur for long term memory and if they result from memory formation, consolidation or retrieval.

### Immunocytochemical Analysis in Hippocampal Nerve Terminals

Hippocampal nerve terminals were purified through a discontinuous Percoll gradient and platted over poly-L-lysine-coated cover-slips for immunocytochemical analysis, using antibodies that were previously validated [22], [23]. Permeabilized nerve terminals were incubated for 1 hour with mouse anti-synaptophysin (1∶200, Sigma-Aldrich), rabbit anti-A_1_R (1∶200, Affinity Bioreagent, Rockford, IL, USA) or goat anti-A_2A_R (1∶200, Santa Cruz Biotechnology, Santa Cruz, CA, USA), and guinea pig anti-vesicular GABA transporters (vGAT, 1∶1000, Calbiochem, San Diego, CA, USA) or guinea pig anti-vesicular glutamate transporters (vGluT1) plus guinea pig anti-vGluT2 (both 1∶1000, Chemicon, Temecula, CA, USA) followed by a 1 hour incubation with AlexaFluor-labelled secondary antibodies (1∶2000, Molecular Probes, Leiden, The Netherlands), which did not yield any signal in the absence of the corresponding primary antibodies. After washing and mounting onto slides with Prolong Gold Antifading (Invitrogen, Eugene, OR, USA), preparations were visualized in a Zeiss fluorescence microscope equipped with a cooled CCD camera and analyzed with MetaFluor 5.0 software [22]. Each coverslip was analyzed by counting three different fields and in each field a total amount of 500 individualized elements, as previously described [23].

### Western Blot Analysis in Hippocampal Membranes

Western blot analysis was carried out as previously described [10], [14], [15], [19], [23] using antibodies against A_1_R (dilution 1∶600), synaptophysin (1∶2000, Sigma-Aldrich), SNAP25 (1∶2000, Sigma-Aldrich), vGluT1 (1∶5000, Chemicon), vGAT (1∶1000, Calbiochem), PSD95 (1∶2000, Upstate Biotechnology, Lake Placid, NY, USA), gephyrin (1∶500, Abcam, Cambridge, UK), MAP2 (1∶200, Santa Cruz Biotechnology) or GFAP (1∶1000, Sigma-Aldrich). The membranes were then re-probed and tested for α-tubulin immunoreactivity to confirm that similar amounts of protein were applied to the gels [10], [14], [15], [19], [23].

### Membrane Binding Assay

The density of A_1_Rs and A_2A_Rs in synaptosomal membranes from the hippocampus was determined using supra-maximal concentrations (6 nM) of either the selective A_1_R antagonist, ^3^H-DPCPX (specific activity of 109.0 Ci/mmol, DuPont NEN, Boston, MA, USA), or the selective A_2A_R antagonist, ^3^H-SCH 58261 (specific activity of 77 Ci/mmol; prepared by Amersham and offered by Dr. E.Ongini, Shering-Plough, Italy), following procedures previously described [14].

### Mouse Brain Histochemistry

The preparation of brain coronal sections (20 µm) was carried out as previously described, after perfusion–fixation with 4% paraformaldehyde in 0.15 mM sodium phosphate buffer, pH 7.4 (for 6 hours) under deep pentobarbital anaesthesia (50 ml/kg, i.p.), followed by a 24 hours post-fixation in the same fixative solution [10], [15]. Neuronal hippocampal morphology was assessed using a cresyl violet staining of Nissl bodies and FluoroJade-C staining was used to evaluate neuronal degeneration and immunohistochemical analysis of dendritic processes [MAP2 staining (1∶500)], of astrocytes [GFAP staining using a Cy3-conjugated anti-GFAP mouse monoclonal antibody (1∶1000, Sigma-Aldrich)] and of nerve terminals [synaptophysin staining (1∶500)] was carried out as previously described [10], [15]. After mounting (Vectashield H-1400, Vector Laboratories, Baptista Marques, Lisbon, Portugal), brain slices were examined under an Axiovert 200 Zeiss fluorescence microscope and digital photomicrographs were obtained at a magnification of either 40× or 63× (oil-immersion lens, numerical aperture of 1.3), with a cooled CCD digital camera, adjusted to these parameters. Images were processed and analysed with the software ImageJ 1.37v (NIH, Bethesda, MD, USA); data from cresyl violet staining was only evaluated qualitatively, whereas the number of degenerated neurons (stained with FluoroJadeC) or the integral of fluorescence (MAP-2, GFAP or synaptophysin staining) were counted in at least 3 regions with 50×50 µm in each section in at least 3 sections per animal. In the case of MAP-2 and synaptophysin staining, it was verified that the secondary antibodies failed to produce any signal in the absence of the primary antibodies, under the experimental conditions used. For all epitopes, we always carried out a parallel analysis by quantitative Western blot analysis, to confirm the trends observed in this semi-quantitative immunohistochemistry analysis.

### Statistics

Results are mean±SEM of n independent animals and significance was considered at P<0.05 using one- or two-way ANOVA followed by the Bonferroni or Newman-Keuls post-hoc tests.

## Results

### Caffeine Attenuated Diabetes-induced Modifications of Body Weight and Glycaemia

To test if caffeine prevented hippocampal alterations induced by diabetes, NONcNZO10/LtJ diabetic mice and control mice were allowed access to caffeine (1 g/L) in the drinking water during 4 months, starting at 7 months of age. Caffeine intake was similar in control and diabetic mice, which displayed similar serum caffeine levels (Table 1) of circa 50 µM that are equivalent to these found in Humans drinking circa 8 cups of coffee daily, *i.e.* a rather high caffeine consumption [20]. Caffeine consumption reduced the weight gain and pre-prandial glycaemia in diabetic mice but failed to modify diabetes-associated hyperinsulinemia (Table 1).

### Caffeine Prevented Diabetes-induced Memory Deficits

**Figure 1:**
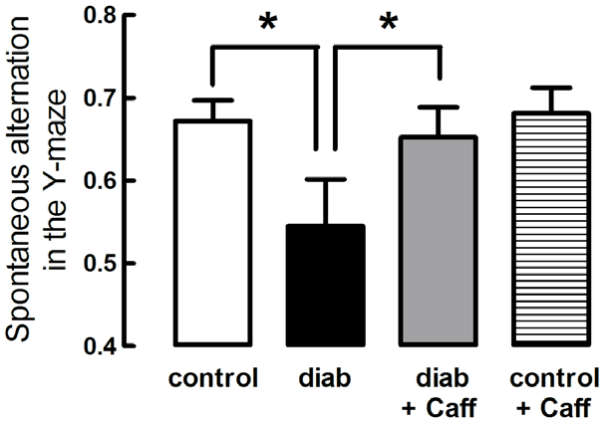
Caffeine consumption prevents memory deficits induced by diabetes. Diabetic mice (black bars) had a reduced spontaneous alternation in the Y-maze at 11 months of age, when compared to controls (white bars), but not if treated with caffeine (grey bars) for 4 months (dark bars), whereas control mice consuming caffeine (striped bars) were similar to controls. In contrast, neither diabetes nor caffeine affected the number of crossings or rearing events in the open-field arena or the total number of entries in the Y-maze arms (not shown). Data are mean±SEM of n = 9–10 mice in each experimental group. *P<0.05 *versus* control.

Behavioural analysis was performed before and after caffeine treatment, at 7 and 11 months of age respectively. Since the Y-maze test is dependent on the exploratory behaviour of mice, locomotion was first evaluated. It was observed that spontaneous locomotion was significantly lower in 11 months compared to 7 months old mice (F_1,65_ = 129.44, P<0.05, n = 8–10); however, neither diabetes (P>0.05) nor caffeine consumption (P>0.05) modified locomotor activity, as indicated by similar number of crossing and rearing events in the open-field arena test and similar number of total entries in the Y-maze arms (data not shown). Spontaneous alternation in the Y-maze task revealed that diabetes reduced the performance of hippocampal-dependent memory at 11 months of age, compared to control mice (P<0.05, n = 8–10; Fig. 1). This diabetes-induced memory impairment was prevented by caffeine consumption (P<0.05 between diabetes and diabetes+caffeine; Fig. 1), which was devoid of effects in control non-diabetic mice (P>0.05 between control and control+caffeine; Fig. 1). Albeit the use of a single behavioural 1 day paradigm only allows a rough estimate of short term memory performance and do not allow determining if memory impairment results from deficits of memory formation and/or consolidation, the data obtained fulfils the targeted goal of supporting that a type 2 diabetic-like condition is associated with memory impairment, as occurs in humans [2]–[4]; additionally, these data suggest that long-term caffeine consumption might prevent diabetes-induced memory impairment.

### Lack of Diabetes-induced Neuronal Degeneration

**Figure 2:**
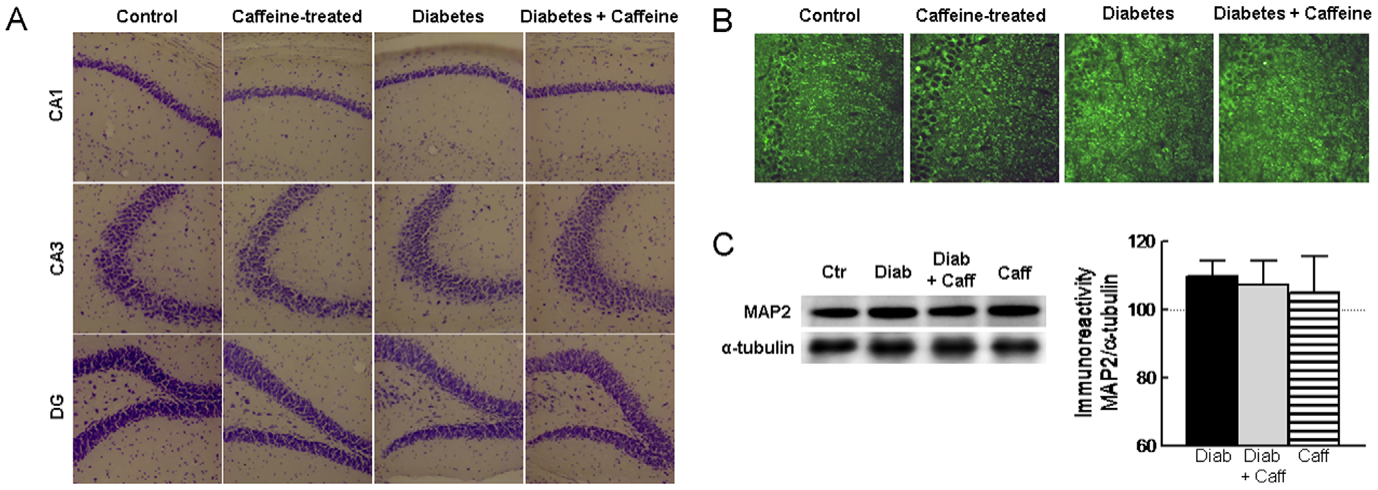
Lack of structural alterations in the hippocampus of diabetic mice. Diabetic NONcNZO10/LtJ mice did not present structural alterations in the hippocampus when evaluating Nissl bodies stained with cresyl violet in the dentate gyrus (DG), CA1 and CA3 regions (A). Neither diabetes nor caffeine consumption affected MAP2 immunoreactivity as evaluated by immunohistochemisty in CA1 area (B) and Western blot analysis (C, n = 6). In C, the immunoreactivity was normalised to that of α-tubulin in the same membranes and calculated as percentage of control. Black, grey and striped bars represent diabetes (Diab), diabetes plus caffeine and caffeine (Caff) groups, respectively. Data in panels (A) and (B) are representative of 4 animals per group and in panel (C) the data are mean±SEM of n = 5–6 mice in each experimental group.

Hippocampal cellular organization and degeneration were evaluated in brain sections stained with cresyl violet and FluoroJade-C, respectively. The qualitative evaluation of cresyl violet staining of the Nissl bodies (Fig. 2A) together with the lack of FluoroJade-C stained cells (data not shown) in the hippocampal formation of the 4 animal groups, supports the lack of diabetes-induced overt neuronal damage in the hippocampus. This was corroborated by the lack of modification of MAP2 immunoreactivity in the hippocampus of diabetic and/or caffeine-treated mice, as assessed quantitatively by Western blot analysis (Fig. 2C) and semi-qualitatively by immunohistochemistry (Fig. 2B).

### Caffeine Attenuated Diabetes-induced Loss of Synaptic Markers

**Figure 3:**
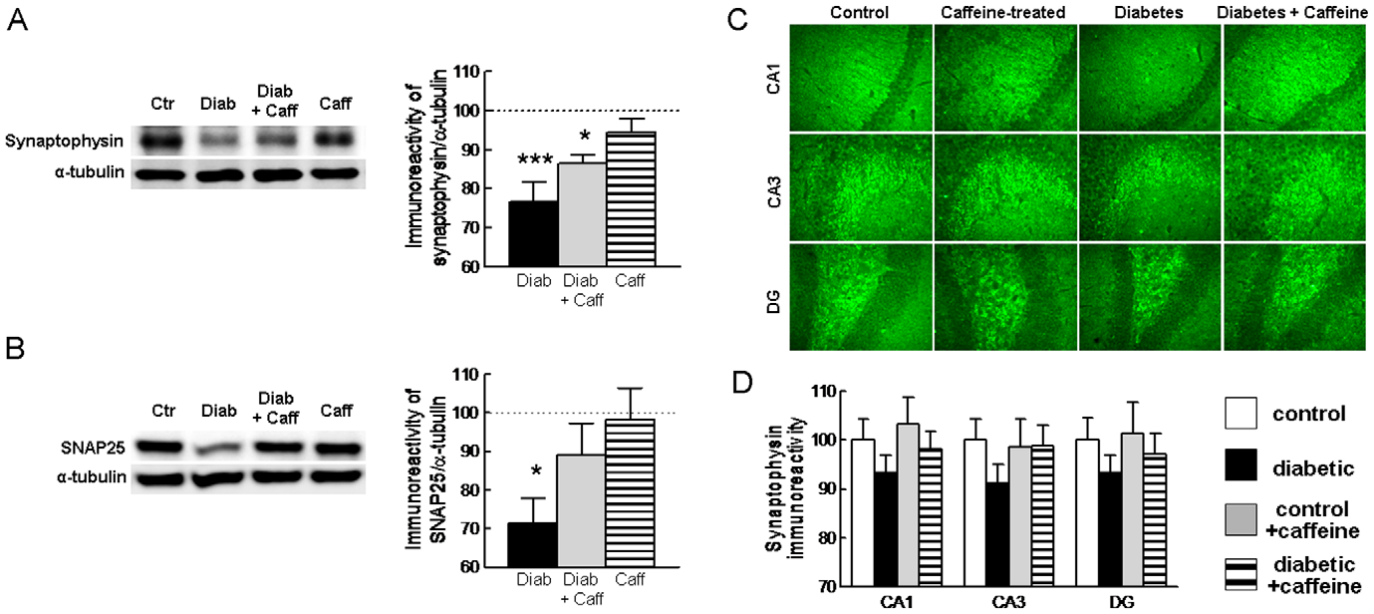
Caffeine attenuates diabetes-induced loss of synaptic markers. Western blot analysis revealed that diabetic mice had a reduced immunoreactivity of both synaptophysin (A) and SNAP25 (B) in hippocampal membranes when compared to controls, which was attenuated by caffeine. The immunoreactivity of synaptic proteins was normalised to α-tubulin in the same membranes and calculated as percentage of control. The black, grey and striped bars represent diabetes (Diab), diabetes plus caffeine and caffeine (Caff) groups, respectively. Panel C shows representative photographs of synaptophysin immunoreactivity, which indicate a tendency (non-significant) for a reduced immunoreactivity in the 3 hippocampal regions in diabetic compared to control mice, as quantified in panel D (% immunoreactivity of control mice). Data in panels (A), (B) and (D) are mean±SEM of n = 5 mice in each experimental group and in panel (C) the data are representative of 5 animals per group *P<0.05, ***P<0.001 *versus* control.

Evidence is accumulating supporting the idea that the deterioration of memory performance is primarily related to synaptic dysfunction and degeneration [24], [25]. Thus, we next tested if NONcNZO10/LtJ mice displayed alterations of two different synaptic proteins integrating the vesicular release machinery, synaptophysin and SNAP25. Western blot analysis (Fig. 3A,B) showed a reduction of the immunoreactivity of synaptophysin (**−**23.3±4.9%, n = 6, P<0.05) and SNAP25 (**−**28.6±6.5%, n = 6, P<0.05) in hippocampal membranes, indicating the occurrence of synaptic degeneration, previously reported to occur in models of type 1 diabetes [14], [15]. Caffeine consumption attenuated the diabetes-induced decrease of SNAP25 (Fig. 3B) and synaptophysin immunoreactivity (Fig. 3A), whereas it was devoid of effects in control mice (Fig. 3A, 3B). Thus caffeine prevents the synaptic deterioration that occurs upon diabetes. Semi-quantitative immunohistochemical analysis of synaptophysin immunoreactivity (Fig. 3C) was not sensitive enough to confirm this reduction in synaptophysin density in the hippocampus; nevertheless, the quantification of synaptophysin immunoreactivity showed a tendency for a systematic reduction in the CA1, CA3 and dentate gyrus of diabetic compared to control or caffeine-treated mice (Fig. 3D).

**Figure 4:**
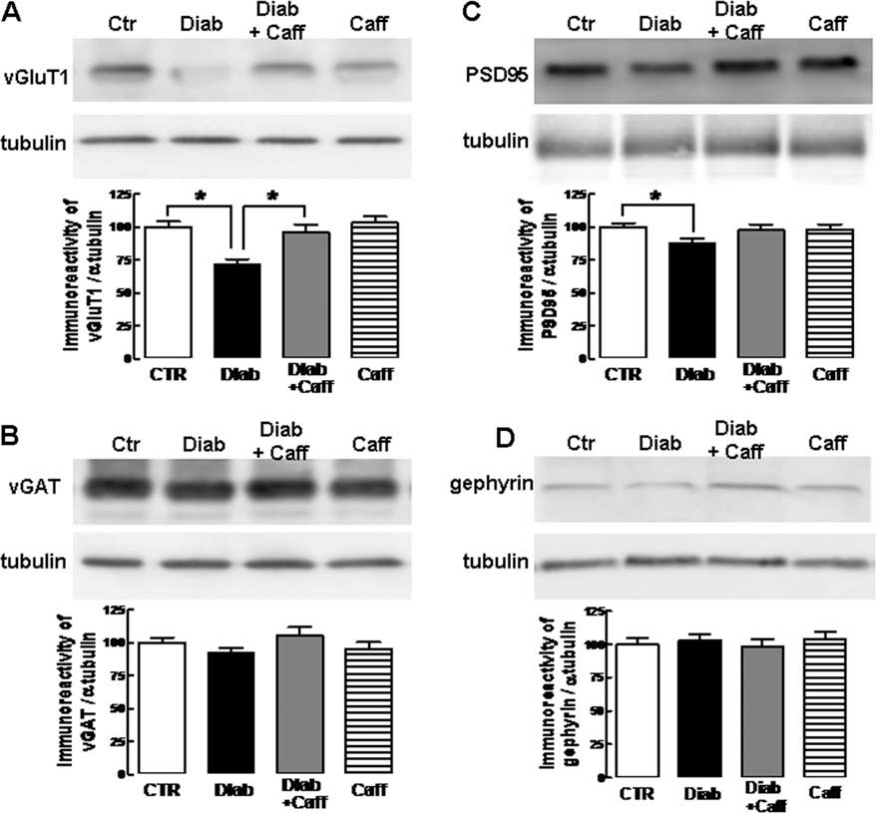
Diabetic mice display a selective loss of markers of glutamatergic rather that GABAergic synapses, which is prevented by caffeine consumption. Type 2 diabetic mice displayed a lower immunoreactivity for markers of glutamatergic (vesicular glutamate transporter type 1, vGluT1, A) rather that GABAergic nerve terminals (vesicular GABA transporter, vGAT, B), and lower immunoreactivity for post-synaptic markers of glutamatergic (pots-synaptic density 95 kDa protein, PSD95, C) rather than GABAergic synapses (gephyrin, D), and all these differences were blunted by caffeine consumption. Open bars represent control mice (CTR), black bars represent diabetic mice (Diab), grey bars represent diabetic mice consuming caffeine (Diab+Caff) and striped bars represent control mice consuming caffeine (Caff). Data are means±SEM of hippocampal membranes from 5 rats per group; * P<0.05 between indicated bars.

**Figure 5:**
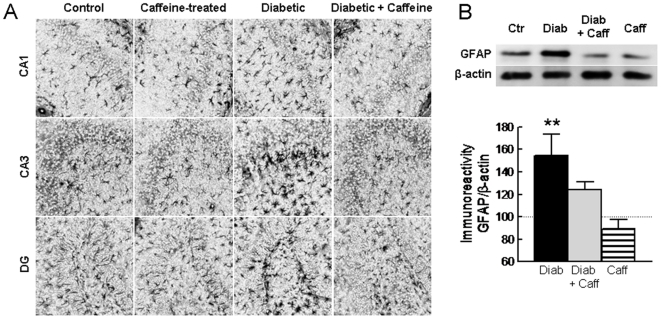
Caffeine preventes astrocytosis caused by diabetes. Panel A shows representative GFAP immunoreactivity photographs of CA1, CA3 and dentate gyrus (DG) showing that diabetic mice displayed increased number of GFAP-positive cells in the hippocampus, but not when consuming caffeine. This was quantitatively confirmed by Western blot (B). GFAP immunoreactivity was normalised to β-actin in the same membrane and calculated as percentage of control. Black, grey and striped bars represent diabetes (Diab), diabetes plus caffeine and caffeine (Caff) groups, respectively. Data in panels (A) and (B) are representative of 4–5 animals per group and in panel (C) the data are mean±SEM of n = 5–6 mice in each experimental group. **P<0.01 *versus* control.

We next investigated if the memory impairment-associated synaptotoxity present in this model of type 2 diabetes mostly affected pre- or post-synaptic components of either glutamatergic or GABAergic synapses. For this purpose, we evaluated by Western blot analysis changes in the density of presynaptic markers of glutamatergic (vGluT1) or GABAergic terminals (vGAT) and of markers of postsynaptic glutamatergic (PSD95) and GABAergic synapses (gephyrin). Figure 4 shows that diabetic mice displayed a reduced immunoreactivity of vGluT1 (**−**28.6±5.3%, n = 5, P<0.05; Fig. 4A) and a preserved immunoreactivity of vGAT (**−**8.2±4.2%, n = 5, P>0.05; Fig. 4B) together with a lower density of PSD95 (**−**12.1±3.7%, n = 5, P<0.05; Fig. 4 C) and preserved levels of gephyrin immunoreactivity (3.2±5.1%, n = 5, P>0.05; Fig. 5D). The consumption of caffeine attenuated the diabetes-induced decrease of vGluT1 (Fig. 4A) and PSD-95 immunoreactivity (Fig. 4C), whereas caffeine consumption failed to modify the immunoreactivity of any of these synaptic markers in control mice (Fig. 4).

### Caffeine Prevented Diabetes-induced Astrocytosis

Semi-quantitative immunohistochemistry of the astrocytic protein GFAP revealed an increased number of GFAP-positive cells in the hippocampus of diabetic mice compared to controls (Fig. 5A). Accordingly, Western blot analysis showed a 57.2±10.4% increase in GFAP immunoreactivity (n = 5, P<0.05) in diabetic mice compared to controls (Fig. 5B). Caffeine consumption reduced this diabetes-induced astrogliosis (Fig. 5), as gauged by the reduction to near control levels of GFAP immunoreactivity and of the number of GFAP-positive cells in the hippocampus.

### Diabetes-induced Modification of the Density of Adenosine Receptors

Caffeine is a mixed antagonist of A_1_R and A_2A_R [5]. The density of cortical A_1_R is 40 times larger than that of A_2A_R [5]; however, noxious brain conditions increase cortical A_2A_R density while decreasing that of A_1_R, which helps explaining why the neuroprotection afforded by caffeine is mimicked by antagonists of A_2A_R but not of A_1_R [11]. Thus, we now tested if the density of A_1_R and A_2A_R in the hippocampus was modified in this mouse model of type 2 diabetes.

**Figure 6:**
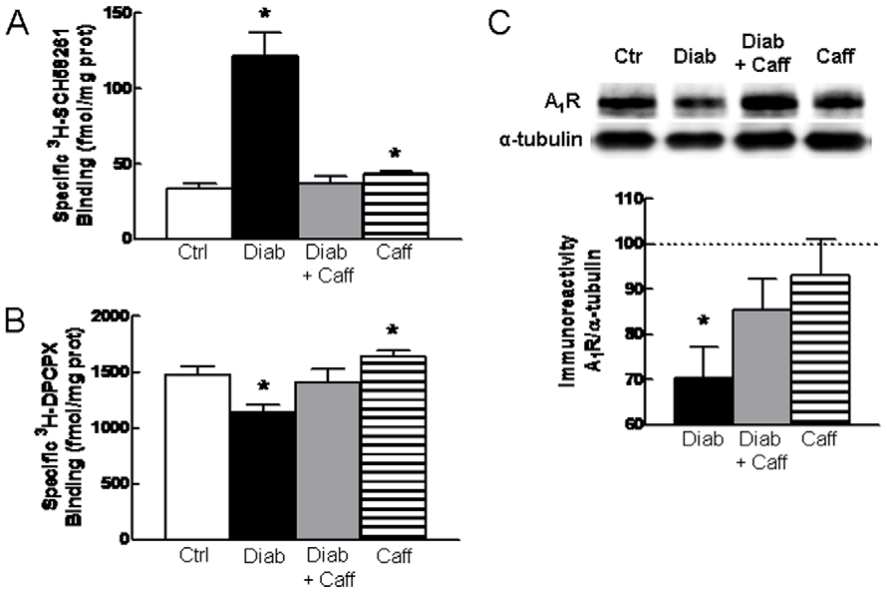
Diabetes increases A_2A_R density and decreases A_1_R density in hippocampal nerve terminals. A_2A_R (A) and A_1_R density (B) were measured as the specific binding of a supra-maximal concentration (6 nM) of the selective antagonists of A_2A_R (^3^H-SCH58261) and A_1_R (^3^H-DPCPX) in membranes from hippocampal nerve terminals of control (open bar), diabetic (black bars), diabetic treated with caffeine (grey bar) and control treated with caffeine (striped bars) (n = 5–6). Panel (C) displays a Western blot comparing A_1_R immunoreactivity in control or diabetic (Diab) mice without or with caffeine (Caff) treatment, as well as the average results of A_1_R immunoreactivity (% of control) in 6 such experiments. *P<0.05 *versus* control.

Binding of the A_2A_R antagonist ^3^H-SCH58261 showed that A_2A_R density increased by 261±43% (Fig. 6A) in diabetic compared to control mice (n = 5, P<0.05). Caffeine consumption also up-regulated A_2A_R to a smaller extent (28.0±3.2%, n = 5, P<0.05) but attenuated the diabetes-induced marked up-regulation of A_2A_R (Fig. 6A).

Binding of the A_1_R antagonist ^3^H-DPCPX showed that A_1_R density decreased by 22.5±1.7% (Fig. 6B) in diabetic compared to control mice (n = 5, P<0.05). Caffeine consumption slightly up-regulated A_1_R (11.0±0.7%, n = 5, P<0.05) and attenuated the diabetes-induced down-regulation of A_1_R (Fig. 6B). Western blot analysis confirmed that diabetic mice display a reduced A_1_R immunoreactivity (**−**29.6±6.7%, n = 6, P<0.05), which was prevented by caffeine (Fig. 6C).

### Diabetes-induced Modification of the Synaptic Distribution of Adenosine Receptors

**Figure 7:**
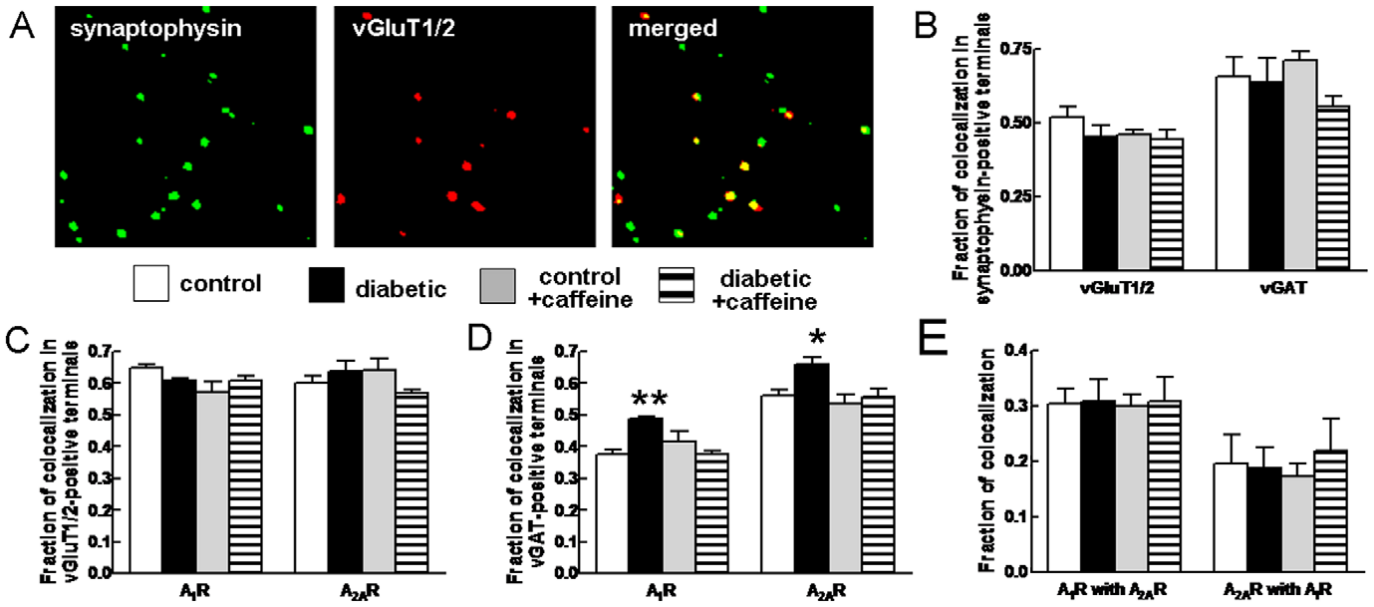
Co-localization of A_1_R and A_2A_R in glutamatergic and GABAergic nerve terminals purified from the hippocampus. Panel (A) exemplifies the double immunostaining performed, here determining the fraction of glutamatergic (vGluT1/2-positive) terminals (synaptophysin immunoreactivity). The fraction of glutamatergic and GABAergic nerve terminals in the hippocampus was similar in the four experimental groups (B). The fraction of glutamatergic terminals containing A_1_Rs or A_2A_Rs was not altered (C) but diabetic mice displayed increased number of GABAergic terminals endowed with A_1_R or A_2A_R (D). The fraction of nerve terminals immunoreactive for both A_1_Rs and A_2A_Rs was similar in the four animal groups (E). White, black, grey and striped bars represent control, diabetes, diabetes plus caffeine and caffeine groups, respectively. Data are mean±SEM of n = 5–6 mice in each experimental group. *P<0.05, **P<0.01 *versus* control.

The distribution of A_1_R and A_2A_R within glutamatergic and GABAergic nerve terminals was evaluated by immunocytochemistry in hippocampal purified nerve terminals (Fig. 7). The fraction of glutamatergic markers (vGluT1/2) or GABAergic marker (vGAT) co-localised with synaptophysin (a marker of all nerve terminals) was not modified either by diabetes or caffeine (Fig. 7B), indicating that the relative density of glutamatergic and GABAergic synapses in the hippocampus does not seem to be affected by either diabetes or caffeine. The immunoreactivity of A_1_R and A_2A_R detected in vGluT1/2-immunopositive terminals was similar in the 4 groups (Fig. 7C), whereas diabetes increased the fraction of vGAT-positive terminals endowed with either A_1_R or A_2A_R immunoreactivity by 30.7±1.3% (P<0.05, n = 4) and 17.9±3.9% (P<0.05, n = 4), respectively (Fig. 7D). This diabetes-induced increase in the fraction of GABAergic terminals equipped with adenosine receptors was prevented by caffeine consumption (Fig. 7D). Finally, we observed no change in the fraction of A_1_R-positive terminals containing A_2A_R, or vice versa (Fig. 7E). This indicates that the distribution of presynaptic A_1_R and A_2A_R in the diabetic hippocampus changes in GABAergic rather than glutamatergic terminals.

## Discussion

The use of a novel age-related animal model of type 2 diabetes, closely mimicking different features of this condition in humans, NONcNZO10/LtJ mice [17], suggest the presence of impaired memory performance, as gauged using the Y maze test; this replicates in this animal model the clinically relevant cognitive deficits present with higher incidence in elderly patients with type 2 diabetes [2]–[4]. Although more detailed behavioural studies are required to define if long term memory is also affected and if memory deficits result from the impairment of memory formation, consolidation or retrieval, the present study reports novel tentative neurochemical modifications underlying this diabetic-induced memory impairment. Thus diabetic mice also displayed a reduced density of hippocampal synaptic proteins when compared to controls but did not display alterations of cellular organization or overt neuronal degeneration in the hippocampus; this was mostly concluded based on Western blot analysis of synaptic markers and confirmed by semi-quantitative immunohistochemistry. Thus, the most evident neuronal modification associated with diabetes-induced memory impairment is confined to synapses rather than involving the loss of neuronal cells. This synaptotoxicity has also been proposed to be a primary and initial feature resulting from different insidious brain insults, namely in mild cognitive impairment [26] and Alzheimer’s disease [10], [24], the neurodegenerative disorder more closely associated with memory impairment; this synaptotoxicity is also an early feature of other conditions known to also involve memory impairment, such as Huntington’s [27] or prion’s diseases [28], HIV infection [29] or schizophrenia [30]. Interestingly, streptozotocin-induced diabetic rats also display a reduced density of synaptic proteins [14], [15], which could be related to the observed spatial memory deficits [1], [15]. Most interestingly, we report a selective reduction of markers of glutamatergic synapses (vGluT1 and PSD95), whereas markers of GABAergic synapses (vGAT and gephyrin) were not statistically affected. This indicates selective changes of glutamatergic rather than GABAergic synapses, in remarkable agreement with recent results indicating that memory-related synaptotoxicity might occur particularly in glutamatergic terminals [19], [31]–[33]. Furthermore, we report that the changes are more evident in presynaptic (vGluT1) rather than post-synaptic markers (PSD95), as occurs in other conditions affecting memory [19], [32]. This suggests that the reduced density of hippocampal synaptic proteins, particularly of glutamatergic terminals, may contribute to the diabetes-induced memory impairment displayed by NONcNZO10/LtJ mice.

Apart from synaptic de-regulation, diabetes-induced memory impairment was also accompanied by astrogliosis; this was mainly concluded based on quantitative Western blot analysis showing an increased GFAP immunoreactivity and was confirmed by the semi-quantitative immunohistochemistry analysis indicating an increased number of GFAP-positive cells in the hippocampus of diabetic mice compared to controls. Upon neuronal damage, astrocytes can become reactive [34] and the hippocampus of diabetic mice displayed such astrocytic hypertrophic cell bodies and increased processes with augmented density of GFAP. Accordingly, it has been proposed that astrogliosis may be the earliest brain responses to hyperglycemia [35]. Astrocytes provide a structural, metabolic and trophic support to neurons, modulating synaptic transmission and plasticity, and acting as sensors and initiators of brain damage [34], [36]. Furthermore, astrocytic dysfunction has been proposed to be of prime importance in the evolution of other brain disorders (reviewed in [37], [38]). This suggests that the observed diabetes-induced astrogliosis in the hippocampus might also contribute for synaptic dysfunction and hampered memory performance.

Although based on correlative analysis, this possible involvement of synaptotoxicity and astrogliosis in the mechanism of diabetes-induced memory impairment is further supported by the main finding of the present work, *i.e.* that long-term caffeine consumption prevented both diabetes-associated memory impairments and diabetes-induced astrogliosis and loss of nerve terminal markers in the hippocampus. This ability of caffeine to prevent diabetes-induced memory deficits is paralleled by the ability of caffeine to prevent memory deficits found in aging [39], [40], Alzheimer’s [9], [21] and Parkinson’s diseases [41], early-life convulsions [19], attention deficits and hyperactivity disorders [42], alcohol consumption [43] and streptozotocin-induced diabetes [15]. Overall, this suggests that caffeine is able to interfere with mechanisms playing a key role in demises of memory dysfunction, acting as a memory stabilizer rather than as a memory enhancer (see [7]). The only known molecular targets for caffeine at non-toxic concentrations are A_1_R and A_2A_R, both antagonised by caffeine [5]. A_2A_R seem to be the main targets of caffeine to prevent memory impairment since: 1) chronic noxious insults enhance the density of A_2A_R while reducing that of A_1_R [11], as now also observed in this model of type 2 diabetes; 2) selective antagonists of A_2A_R rather than A_1_R mimic the beneficial effects of caffeine on afflicted memory performance (reviewed in [6], [7]). Interestingly, A_2A_R antagonism not only abrogates memory dysfunction but also affords robust neuroprotection against brain damage in different animal models of CNS disorders [11], [12], most of which involve initial modifications of synaptic function [24], [25]. This further supports the involvement of synaptotoxicity in memory dysfunction since A_2A_R have a predominant synaptic localization in the hippocampus [44], where they control synaptotoxicity [10], [15], [19], [45], [46], which we now report to be the most evident morphological change found in the hippocampus of diabetic mice. These synaptic A_2A_R undergo a gain of function in noxious brain conditions [11]; accordingly, we now found an over 5 times increase of the density of synaptic A_2A_R in the hippocampus of diabetic mice, as was previously been found to occur in streptozotocin-treated rats [14], [15]. These A_2A_R were not only located in glutamatergic but also in GABAergic nerve terminals. Whereas the role of A_2A_Rs in glutamatergic terminals is in accordance with their ability to control synaptic plasticity [47], the role of A_2A_Rs in GABAergic terminals is still unclear but may be related to the ability of caffeine to control the silence period of cortical firing [48].

Albeit the hypothesis that caffeine controls diabetes-induced memory dysfunction through stabilization of cortical synapses is appealing based on the combined observations that dysfunction and/or loss of nerve terminals is one of the earliest modifications in the course of neurodegenerative diseases [7], [24], [25] and that A_2A_Rs are mostly synaptically-located [44] and control the viability of cortical synapses [10], [15], [19], [45], [46], we also observed that caffeine prevented diabetes-induced astrogliosis. We previously showed that A_2A_R are also up-regulated in glial cells in the injured brain [49] and these astrocytic A_2A_R can control glutamate uptake [50] and the release of NO or interleukins [51]–[52], all effects that can impact on the function and viability of synapses. A recent study showed a direct causal relation between reactive astrocytosis and modifications of GABAergic transmission leading to over-excitability in hippocampal tissue, which was argued to have a metabolic basis [53]. A direct link between neuron-glia metabolism and memory performance has also been provided [54]. Furthermore, the elegant studies of Detlev Boison in animal models of epilepsy and of ischemia have emphasised the central role astrocytic dysfunction in controlling the extracellular levels of adenosine (reviewed in [37]); this adds an additional metabolic dimension to the modifications of purinergic signalling upon brain dysfunction, which was not detailed in the present study. Overall, the observed protective effects of caffeine against diabetes-induced memory dysfunction appear to suggest that both synaptic dysfunction and astrocytic deregulation are expected to be part of the mechanism by which diabetes impacts on memory performance. However, synaptotoxicity and astrogliosis appear to be part of the pathogenic cascade of events of different CNS disorders, and therefore, caffeine consumption might interfere with initial events common to different neurodegenerative brain disorders (see [11]), rather than representing a selective strategy to prevent diabetes-induced memory impairment. It should also be added that caffeine-mediated neuroprotection might also involve other mechanisms responsible for the evolution of neurodegenerative disorders, such as the disruption of the blood brain barrier and neuroinflammation. In fact, caffeine and the antagonism of adenosine receptors has been reported to prevent neuroinflammation [55]–[57] and damage of the blood brain barrier [58] upon brain insults, which prompts considering to test if these changes are present in this animal model of type 2 diabetes and if they are also abrogated upon caffeine consumption.

In summary, the present study provides the first systematic description of a neuropathological phenotype displayed by new model of type 2 diabetes, that more closely mimics the “real-life” condition in the human diabetic population. The MS also provides the first demonstration that the chronic consumption of caffeine prevents memory impairment in type 2 diabetic mice, which are accompanied by a selective loss of presynaptic (mainly glutamatergic markers) and astrogliosis in the hippocampus.
